# Ultrasonication/dynamic maceration‐assisted extraction method as a novel combined approach for recovery of phenolic compounds from pomegranate peel

**DOI:** 10.1002/fsn3.3642

**Published:** 2023-08-21

**Authors:** Hashem Andishmand, Behzad Masoumi, Mohammadali Torbati, Aziz Homayouni‐Rad, Sodeif Azadmard‐Damirchi, Hamed Hamishehkar

**Affiliations:** ^1^ Student Research Committee, Department of Food Science and Technology, Faculty of Nutrition and Food Sciences Tabriz University of Medical Sciences Tabriz Iran; ^2^ Department of Food Science and Technology, Faculty of Nutrition and Food Sciences Tabriz University of Medical Sciences Tabriz Iran; ^3^ Department of Food Science and Technology, Faculty of Agriculture University of Tabriz Tabriz Iran; ^4^ Drug Applied Research Center Tabriz University of Medical Sciences Tabriz Iran

**Keywords:** antioxidant, Box–Behnken design, extraction, optimization, phenolic compounds, plant by‐product

## Abstract

According to recent studies, pomegranate peel (PP) has the potential to be inverted from environmental pollutant waste to wealth due to possessing valuable phenolic compounds at a higher amount compared to edible parts. So far, different types of biological activities such as antimutagenic, antiproliferative, anti‐inflammatory, and chemo‐preventive properties were stated for pomegranate peel extract (PPE) according to chemical composition. In the present research, the probable intensifying effects of two extraction methods and optimum conditions for novel combined method of ultrasonication and dynamic maceration‐assisted extraction of PPE using response surface methodology (RSM) were determined. A Box–Behnken Design (BBD) was employed to optimize three extraction variables, including sonication time (X1), sonication temperature (X2), and stirring speed (X3) for the achievement of high extraction yield of the phenolic compounds and antioxidant activity. The optimized conditions to obtain maximum extraction efficiency were determined as X1 = 70 min, X2 = 61.8°C, and X3 = 1000 rpm. The experimental values were in line with the values anticipated by RSM models, which indicates the appropriateness of the applied quadratic model and the accomplishment of RSM in optimizing the extraction conditions. The results suggest that the extraction of PPE by mix of ultrasonication as a modern method and dynamic maceration as a conventional method could improve its bioactive extractability and the obtained values were higher than any of the methods used. In other words, these two methods together have intensifying effects in increasing extraction efficiency which could further be utilized in food and agricultural industry.

## Graphical abstract

Ultrasonication and dynamic maceration had synergistic effects in increasing extraction yield. Box–Behnken Design and response surface methodology were used for optimization. The pomegranate peel extract can be used as substitute for synthetic antioxidants.

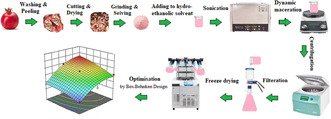

## 1 INTRODUCTION

Pomegranate cultivation area in the world is more than 500,000 hectares with a production of 1.5–2 million tons per year (Balaban et al., 2021; Pawar & Dingre, 2020). The world's major pomegranate producers are India, China, Iran, Turkey, Afghanistan, and the United States, respectively (Ge et al., 2021). In the last decade, due to the therapeutic properties of pomegranate, there has been a considerable increase in the use of its various products such as juice, fresh fruit, jam, and dietary supplements (Chaves et al., 2020; Sharma et al., 2017). On the other hand, the application of industrial processing on pomegranate produces large amounts of side products, especially skin and seeds, which are often discarded as waste or animal feed without any recycling process (Dimitrov et al., 2019; Kaderides et al., 2021). There are various polyphenols such as punicalin, ellagic acid, punicalagin, proanthocyanins, and flavonoids in PP, and the amount of these compounds is at a higher level than the edible parts. The peel of pomegranate contains 50% of its weight (Andishmand et al., 2023; Chen et al., 2020). So far, different types of biological activities such as antimutagenic, antiproliferative, anti‐inflammatory, and chemo‐preventive properties were stated for pomegranate peel extract (PPE) according to chemical composition (El‐Kady et al., 2021; Mastrogiovanni et al., 2019; Viswanath et al., 2019). Furthermore, low toxicity and high safety have been reported by means of in vivo and in vitro studies (Cinar et al., 2021; Hasnaoui, 2022; Mastrogiovanni et al., 2019). Therefore, it seems that PPE has enough potential for shelf life enhancement in food products (Belgacem et al., 2021; Kumar et al., 2022, 2023) and also in formulation of functional foods and nutraceutical supplements (Moussaoui et al., 2021). Ether, chloroform, ethyl acetate, methanol, and ethanol are the organic solvents which could be used for chemical analysis of polyphenols. Between these, only ethanol is food grade and its mixture with water is dominantly used for the recovery of phytochemicals from agri‐food sources (Gaber et al., 2021). The quality and extraction efficiency of the plant extracts depend on the employed extraction procedure and it depends on available facilities and economic efficiency (Javani‐Seraji et al., 2023). To date, various extraction methods, from conventional to modern methods, were also used on pomegranate peel for the phenolic compounds extraction, in which each method having its own advantages and limitations (Javani‐Seraji et al., 2023). Conventional methods because of simplicity and ease of use are popular, but these methods have limitations such as lower extraction efficiency and higher extraction time followed by negative effects on biomolecules because of prolonged exposure (Ameer et al., 2017; Sridhar et al., 2021). Ultrasonication‐assisted extraction method (UAE) could resolve this problem by rupturing of cell wall and enhancing the mass transfer rates by the creation of microcavities, which lead to upper product yields with less extraction time and lower solvent usage (Shirsath et al., 2012). UAE is a modern, simple, cost‐effective, and ecofriendly method (Koraqi et al., 2022; Yusoff et al., 2022). So, it seems that the mix of conventional and modern methods increases extraction efficiency and decreases the negative influence of organic solvents on biomolecules and the environment.

In this research, it has been tried to design and optimize a novel, simple, and profitable combined extraction method based on ultrasonication and dynamic maceration to the improvement of the extraction of phenolic compounds from plant by‐products, which are a rich source of low‐cost natural antioxidants for use in food, drugs, and cosmetics. We chose PP because the plant is readily available and contains abundant phenolic compounds with multiple health and therapeutic effects. It is worth noting that the optimization of extraction of phenolic compounds by ultrasonication‐assisted extraction method has been studied (Foujdar et al., 2020; Kumar & Srinivasa Rao, 2020; Rakshit & Srivastav, 2021; Sharayei et al., 2019; Tabaraki et al., 2012; Živković et al., 2018), but the use of ultrasonic waves only as a pretreatment step to lyse the cell walls and facilitate mass transfer during the step of dynamic maceration (as a conventional method) has not been studied so far.

## 2 MATERIALS AND METHODS

### 2.1 Plant material, standards, and reagents

Pomegranates (Rabab‐e‐Neiriz cultivar) were obtained from a local shop in Tabriz, Iran. Gallic acid, Folin–Ciocalteu, sodium carbonate, phosphate‐buffered saline (PBS), 2,2‐Diphenyl‐1‐picrylhydrazyl (DPPH), and ethanol were acquired from Merck Chemical Co. with analytical grade. Deionized water was utilized for all the solution's preparation steps.

### 2.2 Sample preparation

PP powder samples were prepared according to recent study (Çam et al., 2014) with minor modifications. Pomegranates have been washed twice with deionized water, hand‐peeled, and then cut into 1 cm pieces and dried at 25°C for 5 days. After that, the dried peel pieces were finely ground in an electric mill and the powder was passed across a 325‐mesh screen (dp < 45 μm). The resulting PP powder was collected in a plastic zip‐lock bag and kept out of the light at −18°C until extraction of the phenolic compounds.

### 2.3 Experimental design and statistical model

The initial range of extraction variables was determined by one‐factor tests. Then, a three‐level‐three‐factor Box–Behnken design with five center points (BBD) was applied for optimization according to Bhuyan et al. (2015) with slight modifications. The design consisted of 17 randomized runs. The range of independent variables and their values are shown in Table 1. Optimum extraction conditions were set on responses such as the yield of extract, DPPH scavenging activity, and total phenolic content (TPC). Design‐expert 12 software was applied for experimental design, data analysis, and determination of the optimum conditions. The significance of the factors and the relationship between them (significant differences adjusted at *p* < .05) were analyzed by analysis of variance (ANOVA). Model adequacy was assessed using the coefficient of determination (*R* ^2^), *p‐*values for the model, and tests for lack of fit. The accuracy of the model was confirmed by applying the combined ultrasonication/dynamic maceration‐assisted extraction method at obtained optimal conditions (sonication time, sonication temperature, and stirring speed) to obtain a maximal yield, antioxidant activity, and TPC.

**TABLE 1:** Independent variables and their levels were used in the response surface design.

Independent variables	Levels		
	−1	0	+1
Sonication time (X1) (min)	30	50	70
Sonication temperature (X2) (°C)	50	60	70
Stirring speed (X3) (rpm)	500	750	1000

### 2.4 Extraction process

Powdered PP was blended with ethanol–water 60:40 (v/v) solvent in the ratio of 1:50 g/mL (Živković et al., 2018). The mixture was sonicated with 400 W power and a frequency equal to 28 kHz at different times and temperatures in an ultrasonic bath (Parsonic 30s, Pars Nahand Engineering Co.). After that, the samples were magnetically stirred (MR Hei–Tec) at various speeds for 24 h at 25°C. Then, the samples were centrifuged (Universal 320, Pole Ideal Tajhiz Co.) at 7000 rpm for 20 min, and the supernatant was filtered by vacuum filtration setup using Whatman filter paper No. 1 to remove coarse particles. Ethanol was removed from the extracts with a rotary evaporator (Laborota 4002, Heidolph) at 50°C. After that, the residual PPE was lyophilized (ALPHA 1–4 LD freeze dryer, Martin Christ) at the temperature of −30°C, and a pressure of 0.07–0.1 mbar for 48 h. Finally, the obtained powders were stored in plastic zip‐lock bags at −18°C until further use.

### 2.5 Determination of TPC and yield of extract

The TPC of PPE was determined by the Folin–Ciocalteu method according to recent study (Soltanzadeh et al., 2021). Gallic acid (0–250 μg/mL) was used to calibrate a standard curve. Data were revealed as milligrams of gallic acid equivalents per gram of the dry weight of PPE (mg GAE/g DW PPE) by repetition three times. The yield of extract was determined by the Equation (1):

(1) $$\text{Yield of extract}\;\left(\%\right)=\frac{\text{Weight of}\;\mathrm{PPE}\;}{\text{Weight of}\;\mathrm{PP}\;\text{powder}}\times 100$$

### 2.6 Antioxidant activity measurement

The antioxidant activity of the samples was determined by the DPPH method according to recent study (Peršurić et al., 2020) with some modifications. An alcoholic DPPH solution is reduced the existence of a hydrogen‐donating antioxidant (Andishmand et al., 2016; Soltanzadeh et al., 2022). DPPH reagent was obtained by dissolving 4‐mg DPPH in 100‐mL ethanol 96%. The scavenging activity of sample solutions was done by mixing 1‐mL aliquot of samples (2 mg/mL) with 1 mL of DPPH reagent. A blank solution was also prepared by the exact method, except that ethanol–water 60:40 (v/v) was added instead of the PPE. The samples’ incubation in a chamber away from light for 30 min at a temperature of 25°C was accomplished. The absorbance of the prepared solutions was measured at 517 nm via a spectrophotometer (Ultrospec2000, Pharmacia Biotech). Decreased absorption of DPPH solution indicates high antioxidant activity (Sekowski et al., 2022). The values were calculated by Equation (2):

(2) $$\begin{array}{c} \text{DPPH scavenging activity}\;\left(\%\right)\\ =\frac{\text{Blank absorbance}-\text{Sample absorbance}\;}{\text{Blank absorbance}}\times 100 \end{array}$$

## 3 RESULT AND DISCUSSION

### 3.1 Sonication time impact (in the absence of dynamic maceration)

**FIGURE 1:**
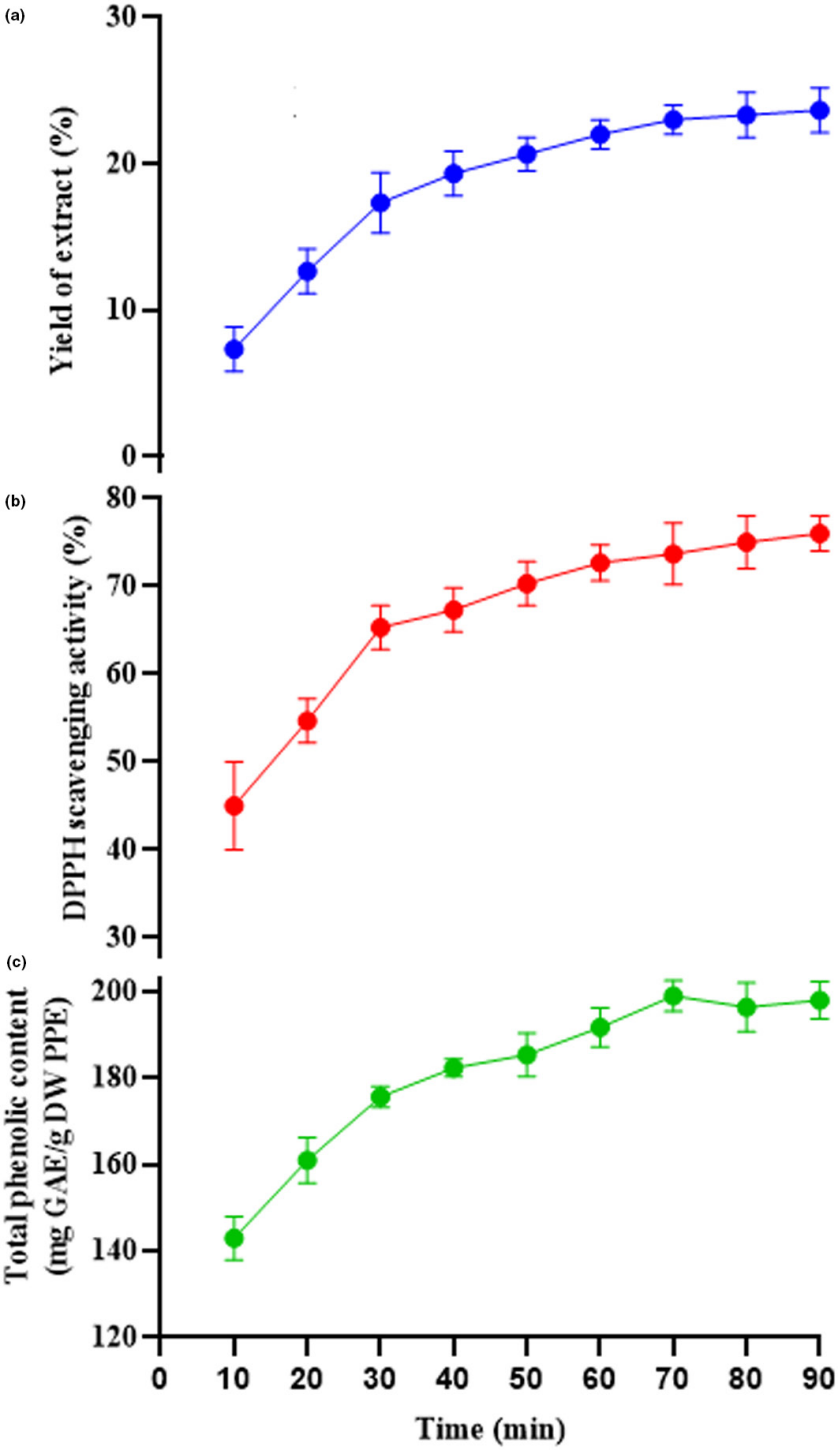
Effect of sonication time on the yield of extract (a), antioxidant activity (DPPH scavenging activity) (b), and total phenolic content (c). DPPH, 2,2‐diphenyl‐1‐picrylhydrazyl; DW, dry weight; GAE, gallic acid equivalent; PPE, pomegranate peel extract.

The yield of extraction can be affected by sonication time (Wu et al., 2021). This could be the result of the solvent penetrating the dried PP powder, dissolving the phenolic compounds, and then diffusing out of the PP (Rajha et al., 2020). The influence of sonication time on the yield of PPE, DPPH scavenging activity, and TPC is displayed in Figure 1. Initially, the sonication time was determined at 10‐min intervals from 10 to 80 min. The other extraction factors were as follows: sonication temperature of 50°C, solvent type of ethanol–water 60:40 (v/v), and the ratio of solid to solvent of 1:50 g/mL. It was observed that almost all three responses increased rapidly as sonication time raised from 10 to 30 min, and then enhanced slowly until 70 min (Figure 1). This indicates that the extraction time of 30–70 min was sufficient for PPE extraction. Thus, the extraction time between 30 and 70 min was considered desirable for the extraction of PPE. Tabaraki et al. (2012) optimized the extraction condition of PPE by ultrasonication‐assisted method. They evaluated the effect of solvent concentration and type, solid‐to‐solvent ratio, temperature, and time of extraction process on the yield of extract, antioxidant activity, and TPC. The optimal conditions for the highest extraction yield were ethanol–water (70/30 v/v) as the solvent, a temperature of 60°C, and an extraction time of 30 min (Tabaraki et al., 2012).

### 3.2 Sonication temperature impact (in the absence of dynamic maceration)

**FIGURE 2:**
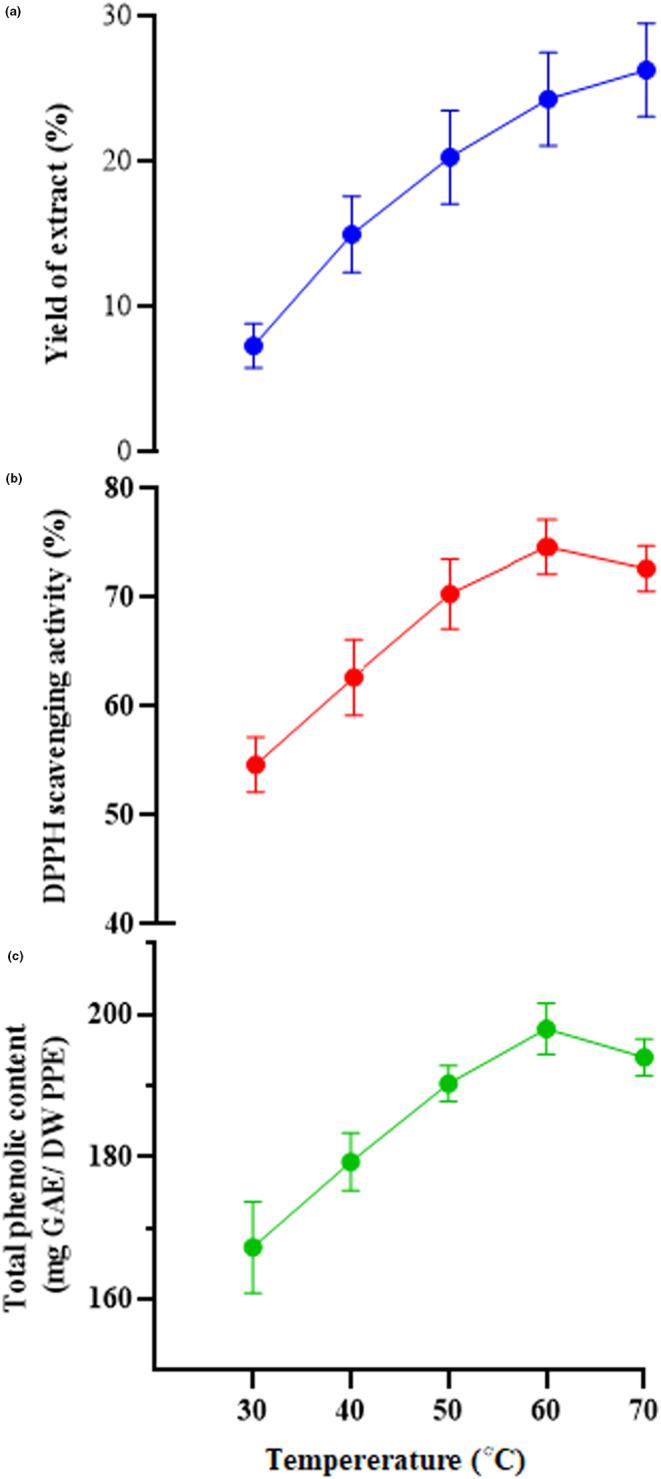
Effect of sonication temperature on the yield of extract (a), antioxidant activity (DPPH scavenging activity) (b), and total phenolic content (c). DPPH, 2,2‐diphenyl‐1‐picrylhydrazyl; DW, dry weight; GAE, gallic acid equivalent; PPE, pomegranate peel extract.

To investigate the impacts of various temperatures on the dependent variables, the extraction was performed at 10°C intervals from 30 to 70°C. The sonication time was adjusted to 50 min. As illustrated in Figure 2, all three responses were enhanced by the extraction temperature increasing from 30 to 60°C. The highest TPC and antioxidant activity were detected at an extraction temperature of 60°C, although they were also high at 70°C. Therefore, an extraction temperature range of 50–70°C was desirable. It seems that phenolic compounds are susceptible to high temperatures, as TPC and antioxidant activity decreased at temperatures above 60°C, and this is agreed with previous investigations. Sumere et al. (2018) optimized the extraction condition of PPE by combining pressurized liquids and probe ultrasonication‐assisted method. They evaluated the effect of solvent type (water, ethanol in three concentrations of 30%, 50%, and 70% v/v), extraction temperature (50–100°C), and some other ultrasound parameters on the yield of extract. The highest TPC was obtained by water solvent extraction at a temperature of 70°C and at temperatures above 80°C, it was significantly reduced (Sumere et al., 2018). Živković et al. (2018), optimized the extraction condition of PPE by ultrasonication‐assisted method. They evaluated the impact of extraction time (10–60 min), ethanol concentration (10%–90%), solid‐to‐solvent ratio (1:10–1:50 g/mL), and extraction temperature (20–80°C) on the yield of extract. Their findings revealed that the extraction of phenolic compounds from PP enhanced with increasing temperature and decreasing solid‐to‐solvent ratio. The yield of extract increased in the early stages and then decreased with the increase of ethanol concentration and extraction time. The optimal conditions for the extraction process were assumed as: ethanol concentration of 59%, extraction temperature of 80°C, extraction time of 25 min, and solid‐to‐solvent ratio of 1:44 g/mL (Živković et al., 2018).

### 3.3 Stirring speed impact (in the absence of ultrasonication)

**FIGURE 3:**
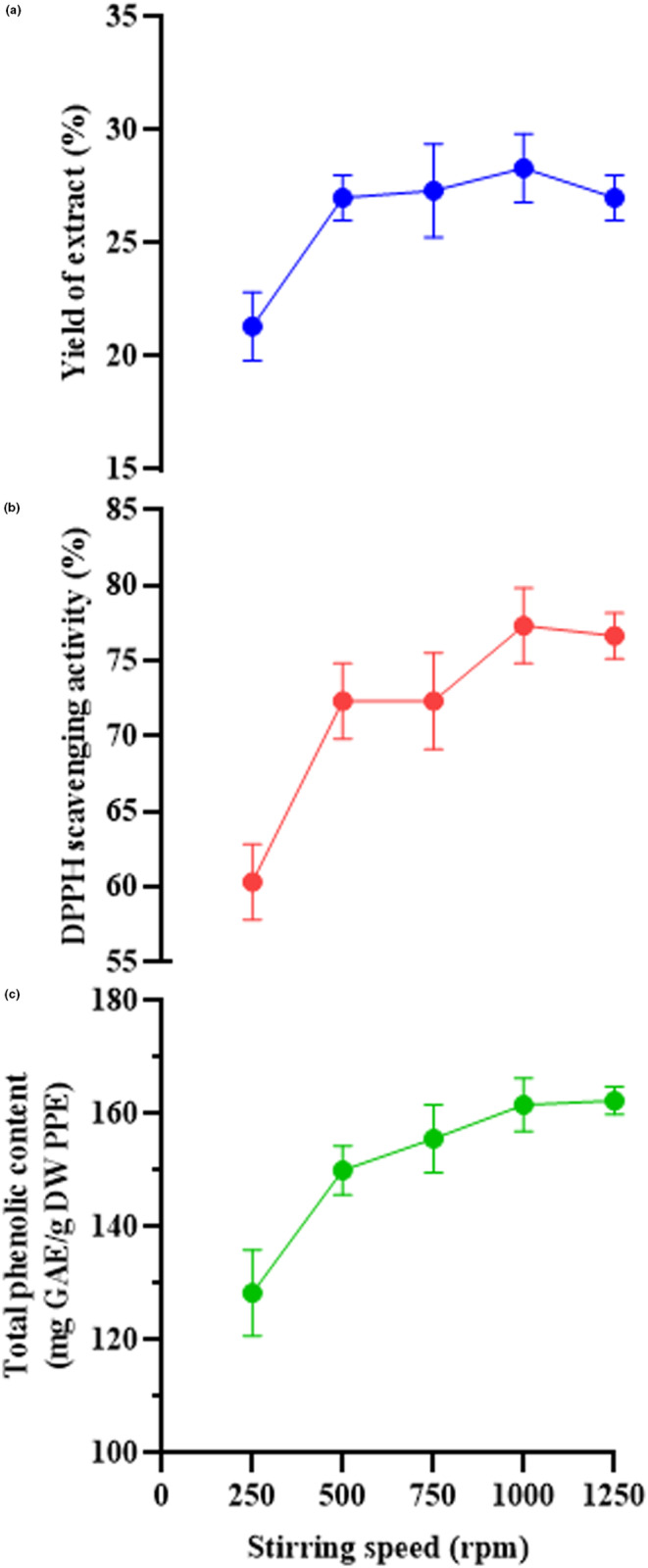
Effect of stirring speed on the yield of extract (a), antioxidant activity (DPPH scavenging activity) (b), and total phenolic content (c). DPPH, 2,2‐diphenyl‐1‐picrylhydrazyl; DW, dry weight; GAE, gallic acid equivalent; PPE, pomegranate peel extract.

Stirring speed is a remarkable element in the extraction of phenolic compounds by the dynamic maceration method (Nekkaa et al., 2021). The change in the speed of the magnetic stirrer leads to the creation of turbulence and eddies, during which the solvent penetrates the plant tissues faster, and the mass transfer rate increases. Moreover, an excessive increase in the stirring speed causes changes in the equilibrium concentration and then the diffusion coefficient (Lampakis et al., 2021; Shishodia et al., 2017). In general, it takes a long time to reach equilibrium with this simple technique but can be modified by combining it with other techniques (Lampakis et al., 2021). In the current investigation, the impact of stirring speed on three responses is shown in Figure 3. First, the stirring speed was set at 250, 500, 750, 1000, and 1250 rpm, while the other extraction factors were assumed as follows: stirring time 24 h, stirring temperature 25°C, solvent type of ethanol–water 60:40 (v/v) and the ratio of solid to solvent 1:50 g/mL. It can be seen that as the stirring speed increased from 250 to 500 rpm, all three responses increased rapidly and then increased slowly until 1000 min (Figure 3). This showed that the stirring speed of 500–1000 rpm is desirable for the production of PPE and this range was chosen as the optimal range for the production of PPE. Nekkaa et al. (2021) extracted bioactive ingredients from *Rhamnus alaternus* leaves via dynamic maceration method. They evaluated the influence of three variables; i.e., stirring speed, extraction time, and the ratio of solid to solvent on TPC and total flavonoid content. The optimum independent variables for extraction process of polyphenols were obtained as: the extraction time of 24 h, the stirring speed of 518 rpm, and the solid‐to‐solvent ratio of 1:10 g/mL (Nekkaa et al., 2021).

### 3.4 Box–Behnken design results

The coded and decoded values of three factors (sonication time, sonication temperature, and stirring speed) and the responses (yield of extract, TPC, and antioxidant activity) of each run are shown in Table 2. TPC of PPE ranged from 186.11 to 283.18 mg GAE/g DW PPE. As illustrated in Table 2, DPPH scavenging activity values ranged from 72.16% to 92.15%, while extraction yields ranged from 24.15% to 38.14%. For the fitted quadratic model, the coefficient of determination (*R* ^2^) for the yield of extract, antioxidant activity, and TPC was 0.9810, 0.9838, and 0.9848, respectively, indicating that only 1.90%, 1.62%, and 1.52% of the total variations were not clarified by the model. The *F*‐value for lack of fit was not significant (*p* > .05), which is a verification for the validity of the model. The value of the adjusted coefficient of determination (adjusted *R* ^2^ of .9566, .9631, and .9653 for the yield of extract, antioxidant activity, and TPC, respectively) also established that the model was exceedingly significant. Moreover, the low values of 2.06, 1.21, and 1.83 of the coefficient of variation (CV) undoubtedly showed significant accuracy and a high degree of reliability of the experimental values. The *p‐*value of the quadratic model was <.0001, indicating that the model was significant. The model proved to be suitable for anticipation in the range of experimental variables.

**TABLE 2:** Box–Behnken design of three variables with their observed responses.

Exp.	X1	X2	X3	Sonication time (min)	Sonication temperature (°C)	Stirring speed (rpm)	The yield of extract (%)	DPPH scavenging activity (%)	TPC (mg GAE/g DW PPE)
1	0	0	0	50	60	750	34.71	86.06	250.69
2	−1	0	1	30	60	1000	29.79	79.83	212.06
3	−1	0	0	30	60	500	29.21	78.11	228.34
4	0	1	0	50	70	500	31.26	79.22	222.95
5	0	0	0	50	60	750	33.94	88.19	259.31
6	0	0	0	50	60	750	33.47	86.12	255.78
7	−1	−1	0	30	50	750	30.04	79.49	238.02
8	1	−1	0	70	50	750	34.69	88.13	266.25
9	1	1	0	70	70	750	34.63	89.26	263.51
10	0	1	1	50	70	1000	33.73	86.23	235.91
11	0	0	0	50	60	750	35.03	87.08	253.86
12	0	−1	−1	50	50	500	34.23	88.91	261.74
13	0	−1	1	50	50	1000	35.88	84.03	250.76
14	−1	1	0	30	70	750	24.15	72.16	186.11
15	1	0	‐1	70	60	500	35.41	90.31	271.82
16	0	0	0	50	60	750	32.91	89.04	265.46
17	1	0	1	70	60	1000	38.14	92.15	283.18

Abbreviations: DPPH, 2,2‐diphenyl‐1‐picrylhydrazyl; DW, dry weight; Exp., experiments; GAE, gallic acid equivalent; PPE, pomegranate peel extract; TPC, total phenolic content; X1–X3, coded factors in Box–Behnken design for sonication time, sonication temperature, and stirring speed, respectively.

The regression coefficient and second‐order analysis of variance of the polynomial models for the yield, DPPH scavenging activity, and TPC of PPE have been brought in Table 3. *p* values were applied to test the significance of the individual coefficient, which in turn can present the pattern of relationship among the parameters. As shown, in most cases, the regression parameters of the surface response analysis of the models, the linear, quadratic, and interaction terms have significant effects (*p* ≤ .001, *p* ≤ .01, or *p* ≤ .05).

**TABLE 3:** Predicted regression model of the relationship between response variables and independent variables (X1, X2, and X3) and analysis of variance for the fitted quadratic polynomial model of extraction.

Source	SS			DF			MS			*F*‐value			*p*‐Value		
	Yield	AA	TPC	Yield	AA	TPC	Yield	AA	TPC	Yield	AA	TPC	Yield	AA	TPC
Model	167.77	453.57	9341.38	9	9	9	18.64	50.40	1037.93	40.19	47.37	50.38	<.0001	<.0001	<.0001
X_1_	110.11	315.76	6062.66	1	1	1	110.11	315.76	6062.66	237.38	296.78	294.29	<.0001	<.0001	<.0001
X_2_	15.32	23.43	1465.84	1	1	1	15.32	23.43	1465.84	33.02	22.02	71.15	.0007	.0022	<.0001
X_3_	6.90	4.05	1.08	1	1	1	6.90	4.05	1.08	14.88	3.80	0.0524	.0062	.0921	.8254
X_1_X_2_	8.50	17.89	604.42	1	1	1	8.50	17.89	604.42	18.32	16.82	29.34	.0037	.0046	.0010
X_1_X_3_	1.16	0.0036	190.99	1	1	1	1.16	0.0036	190.99	2.49	0.0034	9.27	.1585	.9552	.0187
X_2_X_3_	0.1681	35.34	143.28	1	1	1	0.1681	35.34	143.28	0.3624	33.22	6.96	.5662	.0007	.0336
X_1_^2^	14.98	21.65	165.46	1	1	1	14.98	21.65	165.46	32.29	20.35	8.03	.0007	.0028	.0253
X_2_^2^	6.56	32.31	634.81	1	1	1	6.56	32.31	634.81	14.15	30.37	30.81	.0071	.0009	.0009
X_3_^2^	4.31	0.0205	15.22	1	1	1	4.31	0.0205	15.22	9.29	0.0193	0.7388	.0186	.8936	.4185
Residual	3.25	7.45	14.21	7	7	7	0.4639	1.06	20.60						
Lack of fit	0.2102	0.6497	16.14	3	3	3	0.0701	0.2166	5.38	0.0923	0.1274	0.1680	.9604	.9390	.9127
Pure error	3.04	6.80	128.07	4	4	4	0.7592	1.70	32.02						
Cor total	171.02	461.02	9485.59	16	16	16									

Abbreviations: AA, antioxidant activity; DF, Degree of freedom; MS, Mean square; SS, Sum of squares; TPC, total phenolic content.

The high values of *R* ^2^ and adjusted *R* ^2^ indicate that the models effectively represent the experimental results (Table 4). The absence of any lack of fit (*p* > .05) also supported the consistency of all models. The Adeq. Precision determines the ratio of signal to noise. A ratio >4 is suitable. For all three responses, the Adeq. Precision is >25, indicating an acceptable signal and the quadratic model can be used to navigate the design space.

**TABLE 4:** Fit statistics of polynomial quadratic model for the investigated responses from pomegranate peel extracts.

Response fit statistics	Yield of extract	AA	TPC
*R* ^2^	.9810	.9838	.9848
Adjusted *R* ^2^	.9566	.9631	.9653
Predicted *R* ^2^	.9526	.9544	.9517
C.V. %	2.06	1.21	1.83
Adeq. precision	26.7774	25.2460	28.0962

Abbreviations: AA, antioxidant activity; TPC, total phenolic content.

The full models with Equations (3–5) were presented in three dimensions and with contour plots to predict the relationships between the factors and the responses. The second‐order polynomial equations for the reaction surfaces are as follows:

(3) $$\text{Y1}\;\left(g\;\mathrm{PPE}/100\;g\;\mathrm{DW}\;\mathrm{PPE}\right)=34.01+3.71A–1.38B+0.9288C+1.46\mathrm{AB}+0.5375\mathrm{AC}+0.2050\mathrm{BC}–1.89A^{2}–1.25B^{2}+1.01C^{2}$$

(4) $$\text{Y2}\;\left(\text{DPPH scavenging activity}\%\right)=87.30+6.28A–1.71B+0.7113C+2.11\mathrm{AB}+0.0300\mathrm{AC}+2.97\mathrm{BC}–2.27A^{2}–2.77B^{2}+0.0698C^{2}$$

(5) $$\text{Y3}\;\left(\mathrm{mg}\;\mathrm{GAE}/g\;\mathrm{DW}\;\mathrm{PPE}\right)=257.02+27.53A–13.54B–0.3675C+12.29\mathrm{AB}+6.91\mathrm{AC}+5.99\mathrm{BC}–6.27A^{2}–12.28B^{2}–1.90C^{2}$$

where *A*, *B*, and *C* are considered the coded variables of sonication time (min), sonication temperature (°C), and stirring speed (rpm), respectively, from Table 1 and were calculated through the Equation 6:

(6) $$x_{\text{coded}}=[(x_{\text{actual}}–\left(x_{\mathrm{low}}+x_{\text{high}}\right)/2]/\left[\left(x_{\text{high}}–x_{\mathrm{low}}\right)/2\right]$$

### 3.5 Synergistic effects of ultrasonication and dynamic maceration‐assisted extraction methods

When the extraction process was done only by the dynamic maceration method, the extraction efficiency, antioxidant activity, and TPC were obtained in the range of 21%–28%, 58%–78%, and 120–167 mg GAE/g DW PPE, respectively. These values agreed with the results of recent studies on the PPE of the Rabab‐e‐Neiriz cultivar. Soltanzadeh et al. (2021) extracted PPE from Rabab‐e‐Neiriz cultivar by dynamic maceration method. They used methanol–water 80:20 (v/v) in the ratio of 1:5 g/mL solid to solvent, and the extraction process was performed by agitating gently at 25°C for 3 days. The DPPH scavenging activity and TPC were reported to be ~86% and ~ 210 mg GAE/g DW PPE, respectively (Soltanzadeh et al., 2021). It seems that the high amount of TPC and DPPH scavenging activity in their study is related to the solvent type as well as the extraction time (72 h). The hydro‐methanolic solvent, in comparison with hydro‐ethanolic one, possessed higher phenolics extraction capacity but is not food grade. Methanol is highly efficient in the extraction of phenolic compounds than ethanol, even though their polarities are similar. This may be due to the high solvation provided by methanol, perhaps due to the presence of the methyl radical that is shorter than the ethyl radical present in ethanol, causing a higher solvation of phenolic molecules (Boeing et al., 2014).

In another study, Ebrahimnejad et al. (2020) extracted PPE from the Rabab‐e‐Neiriz cultivar by dynamic maceration method. They used distilled water as a solvent in the solid‐to‐solvent ratio of 1:5 g/mL. The extraction process was performed by agitating gently at 25°C for 24 h. The TPC of achieved PPE was reported to be 143 mg GAE/g DW PPE (Ebrahimnejad et al., 2020). Regarding the yield extraction of the two traditional and modern methods, the extraction efficacy of the ultrasonication method was higher than dynamic maceration, which agreed with the earlier reports (Rajha et al., 2019; Turrini et al., 2020). It is worth noting that the extraction with combined ultrasonication/dynamic maceration‐assisted extraction method even provided a much higher phenolic content than the extraction with the ultrasonication method. Hence, it can be concluded that the use of these two methods together has synergistic effects on the efficacy of phenolic compounds extraction. In other words, pretreatment of the samples with ultrasound before the dynamic maceration technique significantly enhanced the yield of extract, DPPH scavenging activity, and TPC up to 38.14%, 92.15%, and 283.18 mg GAE/g DW PPE, respectively.

### 3.6 Effect of process variables

**FIGURE 4:**
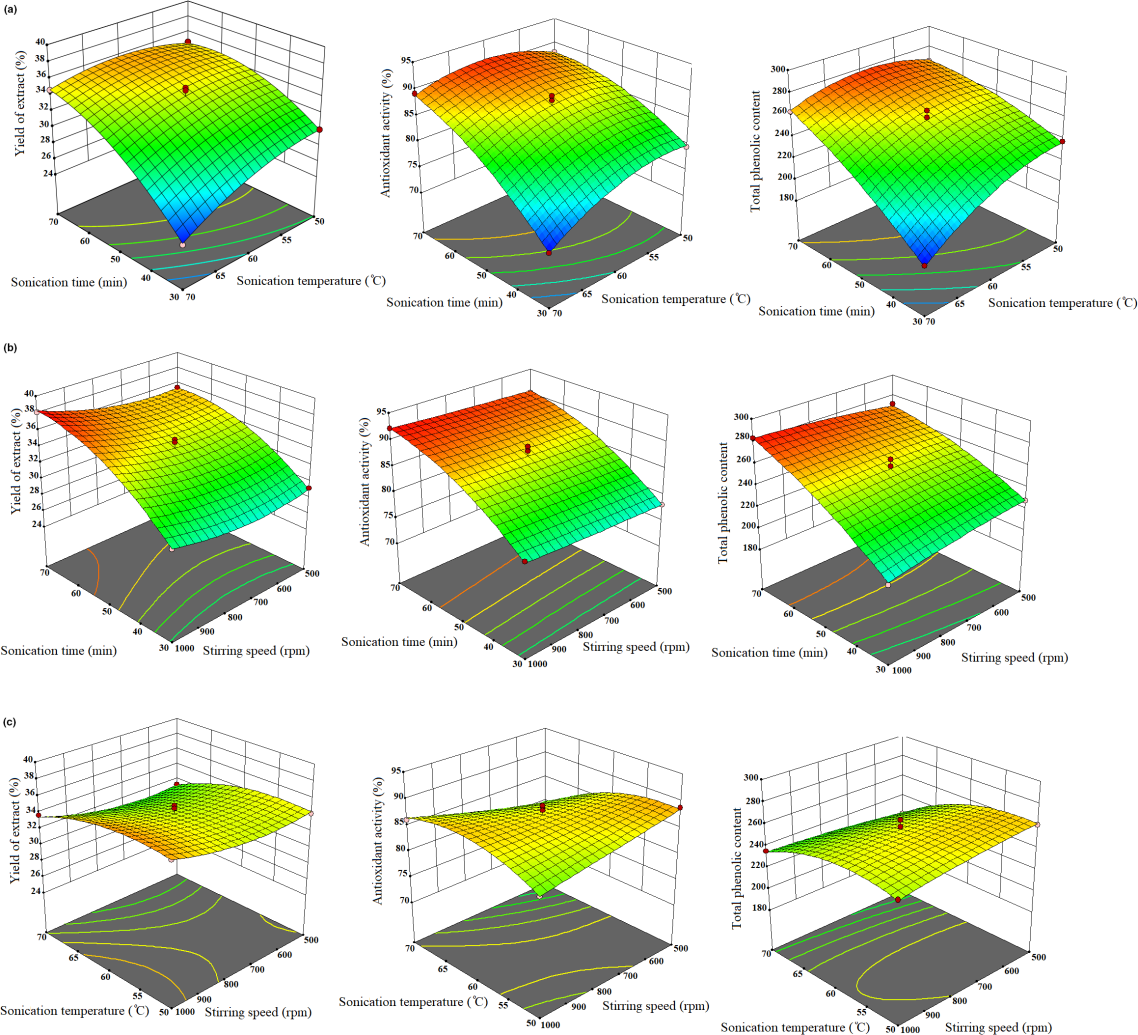
Response surface plots for the effect of (a) sonication time/sonication temperature, (b) sonication time/stirring speed, and (c) sonication temperature/stirring speed on yield of extract, DPPH scavenging activity, and total phenolic content (mg GAE/g DW PPE). DPPH, 2,2‐diphenyl‐1‐picrylhydrazyl; DW, dry weight; GAE, gallic acid equivalent; PPE, pomegranate peel extract.

According to the obtained results, the sonication time had the most significant influence on the yield of extract, TPC, and DPPH scavenging activity. The sonication time showed a quadratic effect on the responses. This effect is expected because, with the increase of sonication time, the phenolic compounds bonded with other components are released and extracted. It has been reported in different studies that the sonication time in the extraction of polyphenols from plant substances directly affects the total amount of polyphenols (Dranca & Oroian, 2016; Mahindrakar & Rathod, 2020; Nadeem et al., 2018). The impacts of sonication time on the yield of extract, antioxidant activity, and TPC at constant stirring speed and sonication temperature are presented in Figure 4a,b, respectively.

Sonication temperature had a considerable influence on the extraction of polyphenols from PP. It had the greatest influence on the TPC. The influence of changing sonication temperature on the yield of extract and DPPH scavenging activity was also statistically significant. The relationship between sonication temperature and sonication time was statistically significant for all three responses, but the relationship between sonication temperature and stirring speed at the constant time was significant only for TPC and antioxidant activity, as shown in Figure 4a,c, respectively. According to reports, heating for half an hour in the temperature range of 52–67°C can lyse plant tissue, affect the integrity of cell walls, and also improve the solubility of polyphenols so that more phenolic compounds are distributed in the solvent (Antony & Farid, 2022; Valdramidis et al., 2011). Stirring speed did not significantly affect TPC and antioxidant activity. The relationship between stirring speed and sonication time only influenced the TPC (Figure 4b), whereas the relationship between stirring speed and sonication temperature influenced both TPC and antioxidant activity (Figure 4c).

Pearson correlation analyses were conducted to define the correlative relationships among the yield of extract, DPPH scavenging activity, and TPC of PPE (Table 5). Consistent with previous reports in polyphenolic‐rich extracts, a strong correlation was observed between DPPH scavenging activity and TPC, which reflected the antioxidant characteristics of PPE. According to Table 5, significant correlations were found between the yield of extracts and their TPC (*r* = 0.903, *p* < .01) and DPPH scavenging activity (*r* = 0.914, *p* < .01). The correlations between the DPPH scavenging activity and TPC (*r* = 0.948, *p* < .01) were also strong. These findings indicated that the yield of extract and TPC are the key determinants associated with the antioxidant activity of PPE.

**TABLE 5:** Correlations between various responses in PPE extraction.

	Yield of extract (%)	DPPH scavenging activity (%)	TPC (mg GAE/g DW PPE)
Yield of extract (%)	1	0.914^a^	0.903^a^
DPPH scavenging activity (%)		1	0.948^a^
TPC (mg GAE/g DW PPE)			1

Abbreviations: DPPH, 2,2‐diphenyl‐1‐picrylhydrazyl; DW, dry weight; GAE, gallic acid equivalent; PPE, pomegranate peel extract; TPC, total phenolic content.

^a^ Correlation is significant at the *p* = .01 level (two‐tailed).

### 3.7 Optimum conditions

The optimal conditions for the extraction of PPE, as determined by the RSM, are shown in Table 6. It is worth mentioning that due to operational limitations, the values closest to the values anticipated by the models and adjustable with the sonicator were selected, and the responses were measured as the maximum actual values. The PPE extraction under optimum conditions was used to investigate the predictive ability of the models. The experimental results obtained under optimal extraction conditions were in line with the results predicted by the models. So, this confirmed the models with proper correlations. The PPE could be substituted with synthetic antioxidants for foods, drugs, and cosmetics. So, the highest yield of extract is suggested for industrial uses. The optimum conditions were the sonication time of 70 min, the sonication temperature of 61.8°C, and the stirring speed of 1000 rpm.

**TABLE 6:** Estimated optimum conditions, predicted, and experimental values of responses under these conditions.

Response variables	Optimum extraction conditions			Maximum values	
	Sonication time (min)	Sonication temperature (°C)	Stirring speed (rpm)	Predicted	Actual
Yield of extract (%)	70	61.8	1000	38.30	38.14
DPPH scavenging activity (%)	70	56.2	515	90.63	90.35
TPC (mg GAE /100 g DW PPE)	70	62.2	1000	282.92	283.18

Abbreviations: DPPH, 2,2‐diphenyl‐1‐picrylhydrazyl; DW, dry weight; GAE, gallic acid equivalent; PPE, pomegranate peel extract; TPC, total phenolic content.

## 4 CONCLUSION

In the present study, we tried to assess the probable synergistic impacts of conventional and modern extraction methods on the yield and the antioxidant activity of PPE. Moreover, the optimum conditions for high recovery of phenolic compounds from PP were obtained using the combined ultrasonication/dynamic maceration‐assisted extraction method. The results indicated that almost all the responses of combined method were significantly higher than each of ultrasonication and dynamic maceration‐assisted extraction methods. So, it seems that these two methods have synergistic effects along together. The impacts of sonication time, sonication temperature, and stirring speed on the extraction of PPE were investigated using RSM. A sonication time of 70 min, a sonication temperature of 61.8°C, and a stirring speed of 1000 rpm were found to be optimal for the highest yield of PPE. The only limitation in the present study was the water bath sonicator device that could only be adjusted at 10°C intervals, and therefore, it was not possible to precisely adjust the temperature provided by the model with the device (e.g., set at 60°C instead of 61.8°C). The parameter that had the highest effect on the yield of extract, DPPH scavenging activity, and TPC was sonication time. Therefore, the ultrasonic/dynamic maceration assisted‐extraction method is a cost‐effective method for producing natural antioxidants. Apparently, PPE has enough potential to be used in the preparation of functional foods and nutraceutical supplements for maintaining health, preventing and treating certain diseases. However, there are some challenges that could limit its applications. Basically, phenolic compounds are prone to degradation due to adverse environmental conditions such as oxygen, light, temperature, pH, etc. Also, ellagitannins could form complexes with salivary glycoproteins and cause an unpleasant taste, which could be prevented by encapsulation techniques (Andishmand et al., 2023). The addition of PPE in an edible matrix for use in food products could also help in the obstruction of lipid oxidation, prevention of microbial contamination, and the improvement of shelf life by satisfying organoleptic properties of food products (Kumar et al., 2022).

## AUTHOR CONTRIBUTIONS

**Hashem Andishmand:** Conceptualization (lead); data curation (lead); formal analysis (lead); investigation (lead); methodology (lead); project administration (lead); resources (lead); software (equal); writing – original draft (lead); writing – review and editing (equal). **Behzad Masoumi:** Formal analysis (equal); investigation (equal); methodology (equal); project administration (equal); resources (equal); software (lead); validation (equal); visualization (equal); writing – original draft (supporting); writing – review and editing (lead). **Mohammadali Torbati:** Funding acquisition (lead); project administration (equal); supervision (lead); validation (supporting); visualization (supporting); writing – review and editing (lead). **Aziz Homayouni‐Rad:** Investigation (equal); methodology (equal); project administration (equal); supervision (equal); validation (equal); visualization (equal); writing – review and editing (equal). **Sodeif Azadmard‐Damirchi:** Conceptualization (equal); data curation (equal); formal analysis (equal); investigation (equal); project administration (equal); supervision (equal); validation (equal); visualization (equal); writing – review and editing (equal). **Hamed Hamishehkar:** Data curation (equal); formal analysis (equal); investigation (equal); methodology (equal); project administration (equal); resources (equal); software (equal); supervision (equal); validation (equal); visualization (equal); writing – review and editing (equal).

## CONFLICT OF INTEREST STATEMENT

There are no conflicts of interest to declare.

## ETHICS STATEMENT

This study does not involve any human or animal testing.

## Data Availability

The datasets analyzed during the current study are available from the corresponding author upon reasonable request.
